# Expression of a symbiosis-specific gene in *Symbiodinium* type A1 associated with coral, nudibranch and giant clam larvae

**DOI:** 10.1098/rsos.170253

**Published:** 2017-05-24

**Authors:** M. Mies, C. R. Voolstra, C. B. Castro, D. O. Pires, E. N. Calderon, P. Y. G. Sumida

**Affiliations:** 1 Oceanographic Institute, University of São Paulo, Praça do Oceanográfico 191, 05508-120 São Paulo, SP, Brazil; 2 Red Sea Research Center, Division of Biological and Environmental Science and Engineering, King Abdullah University of Science and Technology, 23955-6900 Thuwal, Saudi Arabia; 3 Museu Nacional, Universidade Federal do Rio de Janeiro, Quinta da Boa Vista, s/n, 20940-040 Rio de Janeiro, RJ, Brazil; 4 Instituto Coral Vivo, Rua dos Coqueiros, 87-45807-000 Santa Cruz Cabrália, BA, Brazil; 5 Núcleo em Ecologia e Desenvolvimento Socioambiental de Macaé, Universidade Federal do Rio de Janeiro, Av São José do Barreto, 764-27965-045 Macaé, RJ, Brazil

**Keywords:** zooxanthellae, *Tridacna*, scleractinia, sea slug, larval ecology, ATPase

## Abstract

*Symbiodinium* are responsible for the majority of primary production in coral reefs and found in a mutualistic symbiosis with multiple animal phyla. However, little is known about the molecular signals involved in the establishment of this symbiosis and whether it initiates during host larval development. To address this question, we monitored the expression of a putative symbiosis-specific gene (H^+^-ATPase) in *Symbiodinium* A1 *ex hospite* and in association with larvae of a scleractinian coral (*Mussismilia hispida*), a nudibranch (*Berghia stephanieae*) and a giant clam (*Tridacna crocea*). We acquired broodstock for each host, induced spawning and cultured the larvae. *Symbiodinium* cells were offered and larval samples taken for each host during the first 72 h after symbiont addition. In addition, control samples including free-living *Symbiodinium* and broodstock tissue containing symbionts for each host were collected. RNA extraction and RT-PCR were performed and amplified products cloned and sequenced. Our results show that H^+^-ATPase was expressed in *Symbiodinium* associated with coral and giant clam larvae, but not with nudibranch larvae, which digested the symbionts. Broodstock tissue for coral and giant clam also expressed H^+^-ATPase, but not the nudibranch tissue sample. Our results of the expression of H^+^-ATPase as a marker gene suggest that symbiosis between *Symbiodinium* and *M. hispida* and *T. crocea* is established during host larval development. Conversely, in the case of *B. stephanieae* larvae, evidence does not support a mutualistic relationship. Our study supports the utilization of H^+^-ATPase expression as a marker for assessing *Symbiodinium–*invertebrate relationships with applications for the differentiation of symbiotic and non-symbiotic associations. At the same time, insights from a single marker gene approach are limited and future studies should direct the identification of additional symbiosis-specific genes, ideally from both symbiont and host.

## 1. Introduction

Coral reefs are marine coastal environments found in tropical areas, noted for their remarkable biodiversity [1]. This diversity is supported by the complexity of habitats created by the CaCO_3_ structure produced by reef-building corals [2] and by the high primary production performed by symbiotic dinoflagellates, also called zooxanthellae [3–5]. These dinoflagellates (genus *Symbiodinium*) are found in an endosymbiotic association with multiple metazoan and protist phyla [6], being harboured inside the host tissues at high densities, typically 10^10^ cells per m^2^ of coral reef [7].

Before the widespread availability of mainstream molecular biology techniques, a single species of zooxanthella had been described (*Symbiodinium microadriaticum*) and considered pandemic [8,9]. However, through phylogenetic analyses combining ribosomal (nuclear), chloroplast and mitochondrial genes [10–12], *Symbiodinium* dinoflagellates have been proposed to be categorized in nine clades, A–I [13]. Current efforts are concentrating on the formal description of species within the clades [14–16]. Each clade tends to associate with a particular selection of hosts [7,17,18], and in cases of acquisition of heterologous clades, the host typically displays reduced fitness and growth [19–20].

The symbiosis between *Symbiodinium* and their hosts is mutualistic. In exchange for protection, CO_2_, nitrogen and phosphorus [21–23], *Symbiodinium* supplies the host with several organic compounds, including glycerol, glucose, fatty acids and amino acids [24], which may contribute to more than 90% of metabolic requirements of the host [25]. While this metabolite exchange is known for adult hosts, there is scarcity of information for the relationship between *Symbiodinium* and hosts still in their larval stages. The majority of zooxanthellate organisms acquire their symbionts horizontally [26–28], but it is still unknown when and if metabolite exchange initiates during larval development, which may have a crucial impact on the recruitment of coral reef organisms. In addition to these considerations, metabolite exchange and molecular signals are important to ascertain whether a mutualism is in place. Recent studies have sequenced genes in cnidarians that may be symbiosis-specific markers [29–31], but are yet to be tested. While *Symbiodinium* genomes for types within clades A, B and F have been sequenced recently [32–34], only a single symbiosis-specific marker has been suggested to date. This marker is the H^+^-ATPase, a proton pump that transports cations across the cell membrane [35,36] and that is only expressed by *Symbiodinium* engaged in the mutualistic symbiosis [37]. This has been experimentally confirmed comprehensively by Bertucci *et al*. [37] and validated by Mies *et al*. [38]. At present, this gene has only been characterized for *Symbiodinium* A1 [37] and it seems to be little conserved among clades, with a variation in the amount and size of introns, making it more difficult to detect and amplify for *Symbiodinium* species belonging to other clades (M. Mies 2015, unpublished data).

In order to better understand the symbiotic relationship between *Symbiodinium* and coral reef larvae and to assess the general suitability of H^+^-ATPase as a symbiosis marker, we investigated H^+^-ATPase expression by *Symbiodinium* when associated with larvae of three different hosts: (i) *Mussismilia hispida*, a scleractinian coral; (ii) *Berghia stephanieae*, a nudibranch and (iii) *Tridacna crocea*, a giant clam. By investigating these relationships we hope to not only determine whether and when the mutualistic relationship between these organisms is established during larval development, but also to increase our current understanding of coral reef larval ecology, with potential implications for recruitment and dispersal.

## 2. Material and methods

The experiment was designed with the purpose of amplifying the H^+^-ATPase in *Symbiodinium* associated with coral, slug and clam larvae. Therefore, we cultured *Symbiodinium*, spawned and cultured the offspring of the three hosts, offered the cultured *Symbiodinium*, took samples periodically, performed RNA extraction, RT-PCR, cloning and sequencing.

### 2.1. *Symbiodinium* culture

*Symbiodinium* cells (ITS2 type A1) were cultured using the f/2 medium [39], at a temperature of 23°C and a photon flux of 100 µE m^−2^ s^−1^ with a photoperiod of 12 L : 12D. The antibiotics penicillin and streptomycin were added together with the culture medium, at a final concentration of 1.0 and 0.5 g l^−1^, respectively.

### 2.2. Broodstock maintenance and spawning

The *Symbiodinium* hosts selected for this experiment (table 1) were *M. hispida*, a reef-building coral endemic to Brazil with a latitudinal distribution of 2500 km [43]; the stenophagous nudibranch *B. stephanieae* (formerly known as *Aeolidiella stephanieae* and often mistaken for *Berghia verrucicornis*) that feeds exclusively on zooxanthellate anemones of the genus *Aiptasia* [41]; and the smallest species of giant clams, *T. crocea*. All of these hosts naturally house *Symbiodinium* strains belonging to clade A and all of them acquire symbionts horizontally [41,44,45]. All organisms were kept under conditions that simulated tropical reef waters, i.e. temperature at 27°C, specific gravity at 1024 kg m^−3^ and nutrient concentrations near zero. Thirty *M. hispida* colonies (17.5 ± 3.5 cm in approximate diameter) were collected at the Recife de Fora (16°25′ S, 38°59′ W), near the Abrolhos Reefs in northeastern Brazil. Colonies were kept in semi-closed nursery tanks and naturally spawned gamete bundles containing both spermatozoa and oocytes were collected immediately after release. Bundles were dispersed and oocytes fertilized in 60 l tubs and kept for 4 days until planulae had open digestive tracts. Water changes of 90% were performed daily and strong aeration was provided in order to keep the extremely buoyant eggs from becoming trapped in the surface tension. One hundred and thirty broodstock individuals of *B. stephanieae* (1.7 ± 0.3 cm in length) were kept in two 60 l black round tubs in a recirculating aquaria system of 250 l. They were fed 250 individuals of the glass anemone, *Aiptasia* sp. (harbouring *Symbiodinium* A1) and egg masses spawned overnight were collected the next morning. Embryos were then kept for 10 days under strong aeration in order to stimulate the release of veliger larvae [41]. Finally, 10 *T. crocea* broodstock individuals (7.6 ± 0.9 cm in shell length) were maintained in a 350 l recirculating system for 3 months in order to stimulate gamete production [40,46]. They were then induced to spawn with an intragonadal injection of 1.0 ml of a serotonin (5-hydroxytryptamine, 1.0 g l^−1^) solution [47–49]. Fertilization was performed according to Heslinga *et al*. [50] and eggs and, subsequently, trochophore larvae were kept in 60 l black round tubs for 3 days until all larvae attained the veliger stage. Water changes of 50% were performed daily.

**Table 1.** Ecological aspects of the three *Symbiodinium* hosts used in this experiment, including their range distribution, spawning mode, larval size, and mode, stages and total duration of larval development (according to [40–42]). Larval stages in bold denote the stages used in this experiment, at 4, 10 and 3 days post-fertilization, respectively.

host organism	distribution	spawning mode	larvae size at hatching (µm)	larval development mode	stages of larval development	larval development duration
*Mussismilia hispida* (Scleractinia)	tropical Brazil	broadcast spawner	≈300	lecithotrophic	**planula**	≈12 days
*Berghia* *stephanieae* (Gastropoda)	Gulf of Mexico	benthic spawner	≈200	facultative planktotrophic	**veliger**	1–2 days
*Tridacna crocea* (Bivalvia)	tropical Indo-Pacific	broadcast spawner	≈95	planktotrophic	trochophore, **veliger** and pediveliger	≈17 days

### 2.3. Larval cultures, *Symbiodinium* offering and sampling

For each host, larvae were placed in three (replicates) 1.2 l plankton kreisels kept in water baths at 27°C. *Mussismilia hispida* planulae were stocked at 0.8 larva ml^−1^, *B. stephanieae* veligers at 1.0 ml^−1^ and *T. crocea* veligers at 2.0 ml^−1^. *Symbiodinium* A1 was then offered at a final concentration of 10^3^ cells ml^−1^ to all kreisels. At 11 h post-symbiont offering (PSO), a water change of 100% was performed in all kreisels in order to remove *Symbiodinium* cells that had not been acquired. Symbiont acquisition was recorded at this point. Samples of 50, 50 and 250 larvae were taken for *M. hispida*, *B. stephanieae* and *T. crocea*, respectively, at 0, 12, 24, 48 and 72 h PSO. As a positive control for the expression of H^+^-ATPase, tissue (containing symbionts) was retrieved from adult individuals of each host. To confirm that cultured (free-living) *Symbiodinium* do not express H^+^-ATPase, a sample containing 1.0 × 10^6^ cells was obtained. Samples were snap-frozen immediately after collection and kept at −80°C until RNA extraction (see below).

### 2.4. Primer design

In order to confirm the identity of the *Symbiodinium* culture we amplified the internal transcribed spacer 2 (ITS2), using primers designed by LaJeunesse and Trench [51]. Two *Symbiodinium* genes were targeted for this experiment, H^+^-ATPase (Enzyme Commission number 3.6.3.6) and RuBisCO (Ribulose-1,5-bisphosphate carboxylase oxygenase, EC number 4.1.1.39), with the latter chosen as a positive control. Primers for H^+^-ATPase (5′-GCACTTCTTGGGCTTGCTGC-3′ and 5′-ATCTTCCGGGACTCCACCAC-3′) were designed in adjacent regions of two conserved amino acids motifs that are diagnostic for this protein [52], the ATP phosphorylation site (DKTGTLT) and the ATP binding site (TGDGVND). The design was based on conserved regions from the alignment of several sequences obtained from transcriptomes and expressed sequence tags of multiple *Symbiodinium* clades and other dinoflagellates [53–61]. The RuBisCO primers (5′-ACCGGCGTGGGCAAGCTGTTCTCT-3′ and 5′-TGGGAGTGGTCTGCTTCATG-3′) were taken from Bertucci *et al*. [37].

### 2.5. RNA extraction and RT-PCR reaction

Total RNA was extracted from all samples, including the cultured *Symbiodinium* and the tissues from coral, nudibranch and giant clam broodstock. Samples were macerated with a mortar and a pestle, and TriReagent (Ambion) was used for the extraction with modifications suggested in Rosic and Hoegh-Guldberg [62]. Extracted RNA was then treated with the Turbo-DNA-Free kit (Ambion) and the cDNA was generated using the SuperScript First Strand Synthesis III kit (Invitrogen). Approximately 50 ng of cDNA was used in the RT-PCR in a reaction volume of 25 µl, with final concentrations of 2.0 mM MgCl_2_, 0.2 mM dNTPs, 0.15 mM for both forward and reverse primers and 0.04 units µl^−1^ of GoTaq DNA polymerase (Promega). Cycling conditions for H^+^-ATPase and RuBisCO were the following: 3 min at 94°C, 35 cycles of 1 min at 94°C, 1 min at 54°C and 1.5 min at 72°C, and termination at 72°C for 5 min. Cycling conditions for the ITS2 were according to LaJeunesse and Trench [51].

### 2.6. Cloning and sequencing

The amplicons produced were separated on 0.8% agarose, band-purified using the Nucleospin Extract II kit (Macherey-Nagel) and ligated into pGEM T-Easy vector (Promega). Vectors were transformed into electrocompetent cells (DH10B) according to standard practices described in Sambrook *et al*. [63]. Plasmid minipreparations, also according to Sambrook *et al*. [63], were performed for each RT-PCR reaction and sequenced on a 3130XI sequencer using T7 vector primer.

### 2.7. Phylogenetic analysis

Nucleotide sequences related to both H^+^-ATPase and RuBisCO sequences produced in this experiment were retrieved from the National Center for Biotechnology Information (NCBI) using the BLAST algorithm [64]. Maximum-likelihood phylogenies for both genes were generated in MEGA5 [65] using the optimal model of nucleotide substitution (default settings) and a bootstrap of 1000 replicates.

## 3. Results

**Figure 1.**
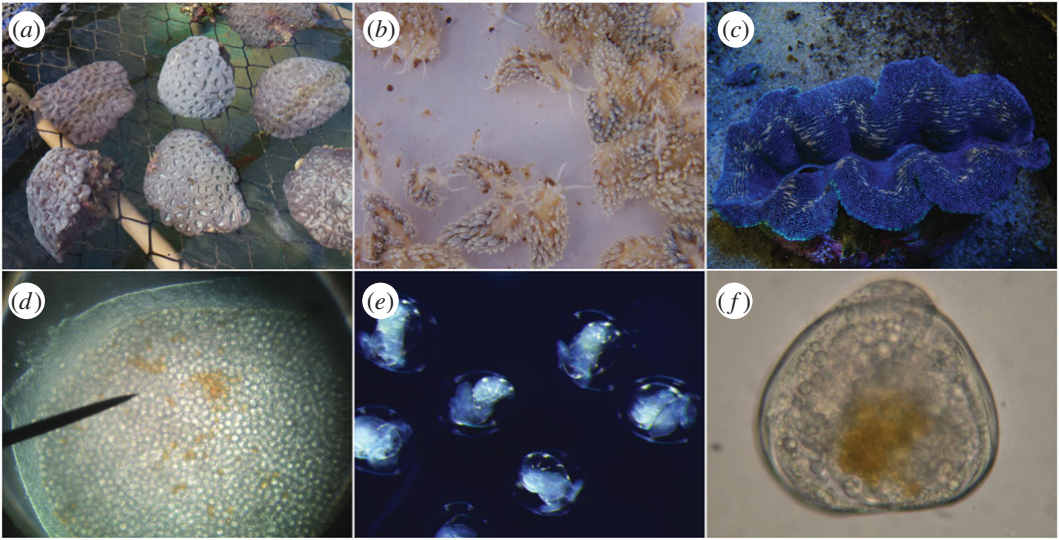
Host broodstock and larvae used in the experiment. (*a*) *Mussismilia hispida* colonies collected at Recife de Fora, (*b*) *Berghia stephanieae* spawning individuals (note brownish area in the cerata, harbouring *Symbiodinium* cells captured from the anemone *Aiptasia* sp.), (*c*) *Tridacna crocea* broodstock clam, (*d*) *Mussismilia hispida* planula after acquiring multiple *Symbiodinium* A1 cells, (*e*) *Berghia stephanieae* veliger larvae immediately before hatching and (*f*) *Tridacna crocea* veliger larva with *Symbiodinium* A1 cells inside the digestive tract.

More than 99% of all host larvae acquired symbionts. The number of symbionts acquired varied greatly among hosts. Symbiont acquisition per planula larva of *M. hispida* was 194.5 ± 31.6 cells, while *B. stephanieae* and *T. crocea* veligers acquired 19.2 ± 5.0 and 36.6 ± 6.5 cells, respectively. *Tridacna crocea* veligers kept the symbionts in the digestive tract throughout the duration of the experiment, while *M. hispida* planulae seemed to move them from the gastrovascular cavity to different areas in the endoderm (figure 1). Stereomicroscopic observations showed that *B. stephanieae* larvae digested the symbionts (*Symbiodinium* cells were degraded, presented ruptured walls and missing or deformed organelles) and did not move them to specialized tissues or cells. Many *B. stephanieae* individuals underwent metamorphosis after 48 h PSO and all of them had become juveniles at 72 h PSO.

**Figure 2.**
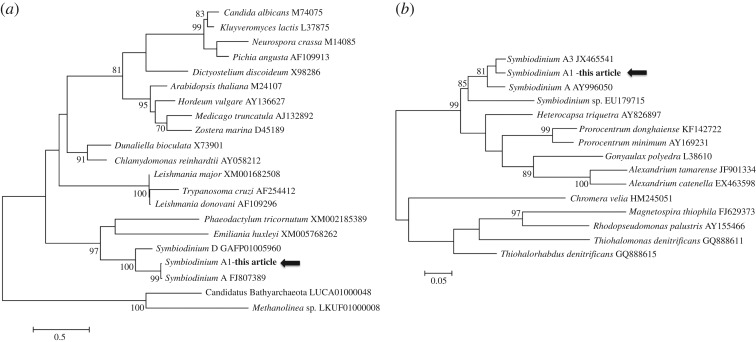
Phylogeny of (*a*) H^+^-ATPase and (*b*) RuBisCO genes of *Symbiodinium* A1 in this experiment. Trees were constructed using maximum-likelihood analysis and 1000 bootstrap replicates; only values above 70 are shown. Accession numbers are from the NCBI database.

The ITS2 amplification confirmed that the *Symbiodinium* cells belonged to type A1. The amplicons produced for H^+^-ATPase and RuBisCO genes had a size of 460 and 430 bp, respectively (GenBank accession numbers KY483989-997). The BLAST searches and phylogenetic trees (figure 2) confirmed that the sequences obtained belong to *Symbiodinium* A1. All H^+^-ATPase and RuBisCO sequences obtained were 99 and 100% identical in pairwise comparisons, respectively. Phylogenetic analyses (figure 2) and BLAST results confirm that the targeted genes were amplified and belonged to *Symbiodinium* A1. The H^+^-ATPase sequences were 99% identical to *Symbiodinium* A1 (GenBank accession number FJ807389) and RuBisCO sequences were 95% identical to *Symbiodinium* (GenBank accession number JX465541).

Free-living *Symbiodinium*, as expected, did not express the H^+^-ATPase. Broodstock tissue containing symbionts from both *M. hispida* and *T. crocea* did express the H^+^-ATPase, while tissue from *B. stephanieae* did not. Out of the three replicated larval cultures for *M. hispida*, the H^+^-ATPase gene was expressed in only one replicate, at 72 h PSO (table 2). This gene was not expressed by any *B. stephaniae* larval replicates, at any time. For *T. crocea*, one of the replicates expressed the H^+^-ATPase at 24, 48 and 72 h PSO, the second replicate only at 24 and 72 h PSO and the third replicate did not express the gene. The RuBisCO gene was expressed for all broodstock tissue and larval samples, as well as for the free-living *Symbiodinium* in culture.

**Table 2.** Expression of H^+^-ATPase by *Symbiodinium* A1 acquired by *Mussismilia hispida* (scleractinian coral), *Berghia stephanieae* (nudibranch) and *Tridacna crocea* (giant clam) larvae over 72 h after acquisition. Expression for cultured *Symbiodinium* A1 (free-living) and tissue of host broodstock was also monitored. All samples exhibited expression of RuBisCO (positive control). +, positive expression; −, no expression; n.a., not applicable.

Sample	Control samples	0 h	12 h	24 h	48 h	72 h
*Symbiodinium* A1 culture	−	n.a.	n.a.	n.a.	n.a.	n.a.
*Mussismilia hispida*						
broodstock tissue	+	n.a.	n.a.	n.a.	n.a.	n.a.
larvae replicate 1	n.a.	−	−	−	−	−
larvae replicate 2	n.a.	−	−	−	−	+
larvae replicate 3	n.a.	−	−	−	−	−
*Berghia stephanieae*						
broodstock tissue	−	n.a.	n.a.	n.a.	n.a.	n.a.
larvae replicate 1	n.a.	−	−	−	−	−
larvae replicate 2	n.a.	−	−	−	−	−
larvae replicate 3	n.a.	−	−	−	−	−
*Tridacna crocea*						
broodstock tissue	+	n.a.	n.a.	n.a.	n.a.	n.a.
larvae replicate 1	n.a.	−	−	+	+	+
larvae replicate 2	n.a.	−	−	+	−	+
larvae replicate 3	n.a.	−	−	−	−	−

## 4. Discussion

The establishment of a mutualistic symbiosis is a process that requires the successful completion of many steps, such as symbiont acquisition, transfer to specialized cells/tissues, metabolite and/or favour exchange and long-term persistence [66]. However, very little is known about the biochemical and molecular mechanisms involved in the establishment of this relationship. The only difference reported thus far is the expression of H^+^-ATPase, a symbiosis-specific gene [37]. Arguably having multiple marker genes would strengthen the here-conducted study and further support our conclusions. In particular, it would be valuable to have symbiosis marker genes for the symbiont and host. However, although a number of studies have investigated differential expression in marine invertebrate hosts with and without *Symbiodinium* [30,67–71], few have attempted to suggest symbiosis marker genes that can be reliably used to assess symbiotic states. Consequently, we focused on assessing H^+^-ATPase as an indicator for establishment of a symbiosis relationship. This gene was only found to be expressed in symbiotic relationships as shown by Bertucci *et al*. [37] and validated by Mies *et al*. [38]. The protein coded for by the H^+^-ATPase is responsible for several reactions, particularly in generating proton gradients across the plasma membrane and dehydrating HCO_3_^−^ [22,35,72]. This gene is also present in other photosynthetic eukaryotes such as the angiosperm *Arabidopsis thaliana* [73] and the planktonic green alga *Platymonas viridis* [74]. While many studies have observed symbiont acquisition by metazoan larvae [19,27,30,75–80], very few tested metabolite exchange or symbiosis-specific molecular signals [38,81]. For that purpose, we decided to investigate whether larvae of several marine invertebrate coral reef taxa express H^+^-ATPase as a result of *Symbiodinium* acquisition.

While only few and nearly undetectable differences were found in comparative analyses of the transcriptomes of symbiotic and aposymbiotic coral larvae [68,70,71], we did find expression of H^+^-ATPase in *Symbiodinium* acquired by the larvae of the coral *M. hispida*. This expression was found in only one of the triplicates, at 72 h PSO, however. We argue that monitoring the expression of this gene for a longer period of time during the larval development would probably give a broader view on the establishment of this mutualistic symbiosis. Nevertheless, our results do show that *Symbiodinium* A1 and coral larvae may engage in symbiosis. In particular, these findings relate to reports that *Symbiodinium* A1 acquired by *M. hispida* larvae produce a higher amount of fatty acids and present lower bleaching rates than most of the other *Symbiodinium* clades [82,83].

The nudibranch *B. stephanieae* is a valuable product in the marine ornamental trade [84]. In our experiments with this species, the expression of H^+^-ATPase was not detected in any of the larval samples, and, more importantly, neither in the broodstock tissue sample. In fact, host larvae were digesting the symbionts. Despite evidence that *Symbiodinium* engages in a mutualistic relationship and translocates photosynthetically fixed carbon to the nudibranch *Pteraeolidia ianthina* [85], this does not seem to be the case for *B. stephanieae*. While adult specimens do host *Symbiodinium* cells in the cerata, there is much evidence against mutualism in the case of this species, especially from the *Symbiodinium* perspective: *B. stephanieae* are nocturnal organisms and remove the symbionts from the *Aiptasia* anemone (which is found in sunlit areas) and later defecates the non-motile *Symbiodinium* cysts after 3–6 days from acquisition [86]. This not only deprives *Symbiodinium* of light, but also renders it an easy prey in the benthos. Furthermore, it has been reported that some nudibranchs sequester *Symbiodinium* from their prey, but may not engage in symbiosis [87,88]. Therefore, the association between *B. stephanieae* and *Symbiodinium* does not seem to fit the requirements for a mutualistic symbiosis. Regardless, this example supports that the expression of H^+^-ATPase is not an endocytosis signal.

In the case of *T. crocea*, an important commodity for both the food and aquarium trade [89], many studies based on morphological examinations suggested that symbiosis was not established until metamorphosis [27,90], when symbionts migrated to post-metamorphic diverticulae called zooxanthellal tubular system [91]. However, studies show that veliger larvae grow faster and have increased survival if symbionts are available [27,45]. Our results show that *Symbiodinium* cells in two of the three replicates of *T. crocea* larvae expressed H^+^-ATPase, in agreement with the findings of Mies *et al*. [38] for *T. maxima*. Similarly to that reported for coral larvae, *Symbiodinium* acquired by *T. crocea* veliger also seem to produce more fatty acids and to be more resistant to bleaching [82,83]. However, the expression of H^+^-ATPase was intermittent for one of the replicates (table 2). While there is very little information available in the literature to explain why this would happen, we argue it may be related to the significant changes associated with the circadian rhythm in *Symbiodinium* and other dinoflagellates, which include variations in metabolite production, gene expression, behaviour and morphology [92–94]. Nonetheless, this event reinforces that modulation of the expression H^+^-ATPase requires further investigation.

It is important to note that the host organisms selected for this experiment, and particularly their larval ecology, are very different (table 1). Scleractinian coral larvae are known lecithotrophs and go through metamorphosis without any exogenous feeding [95], while giant clam veligers are planktotrophs and must feed before attaining the juvenile stage [27,45,96]. Based on H^+^-ATPase expression, our findings argue that giant clam larvae establish symbiosis with *Symbiodinium* earlier than coral larvae, which could point to their higher need of exogenous nutrition. While the nudibranchs also produce lecithotrophic larvae, they are facultative planktotrophs and *Symbiodinium* seems to be more of a prey item than a symbiont, as H^+^-ATPase was never expressed and *Symbiodinium* cells were digested.

While our experiments may contribute to the knowledge on the state of the symbiotic relationship between *Symbiodinium* and coral reef larvae by means of H^+^-ATPase expression, there is still an overwhelming lack of marker genes for *Symbiodinium*. This hinders functional genomics studies [97]. Investigating comparative differential gene expression in the free-living and coccoid (symbiotic) stages is crucial for further understanding the relationships between coral reef organisms and their symbionts. As an example, the expression of H^+^-ATPase may be tied to the non-motile coccoid life stage of *Symbiodinium*. Interestingly, the shift from free-living to coccoid stage has been shown to be chemically stimulated by lectin produced by the coral host [98]. Regardless, our results support the application of H^+^-ATPase gene expression as a molecular symbiosis-specific marker for *Symbodinium*–invertebrate associations. This gene may be used for distinguishing between symbiotic and non-symbiotic associations (e.g. the case of the nudibranch *B. stephanieae*). Our findings may also provide insights for coral reef restoration and aquaculture protocols [99,100], as early symbiont acquisition and mutualism establishment may improve survival and metamorphic competence.

## Data accessibility

All the sequences produced in this article were deposited in the GenBank database, under accession numbers KY483989--997.
